# Affective Neurofeedback Under Naturalistic Conditions: A Mini-Review of Current Achievements and Open Challenges

**DOI:** 10.3389/fnrgo.2021.678981

**Published:** 2021-05-24

**Authors:** Lucas R. Trambaiolli, Abhishek Tiwari, Tiago H. Falk

**Affiliations:** ^1^Basic Neuroscience Division, McLean Hospital–Harvard Medical School, Belmont, MA, United States; ^2^Institut National de la Recherche Scientifique, University of Quebec, Montreal, QC, Canada

**Keywords:** neurofeedback, emotion regulation, virtual reality, naturalistic, in-the-wild, brain-computer interfaces

## Abstract

Affective neurofeedback training allows for the self-regulation of the putative circuits of emotion regulation. This approach has recently been studied as a possible additional treatment for psychiatric disorders, presenting positive effects in symptoms and behaviors. After neurofeedback training, a critical aspect is the transference of the learned self-regulation strategies to outside the laboratory and how to continue reinforcing these strategies in non-controlled environments. In this mini-review, we discuss the current achievements of affective neurofeedback under naturalistic setups. For this, we first provide a brief overview of the state-of-the-art for affective neurofeedback protocols. We then discuss virtual reality as a transitional step toward the final goal of “in-the-wild” protocols and current advances using mobile neurotechnology. Finally, we provide a discussion of open challenges for affective neurofeedback protocols in-the-wild, including topics such as convenience and reliability, environmental effects in attention and workload, among others.

## 1. Introduction

Neurofeedback is a sub-area of brain-computer interfaces (BCIs), in which the subject is trained to achieve voluntary control of the ongoing neural activity in brain regions or circuits (Sitaram et al., 2017; Watanabe et al., 2017; Thibault et al., 2018). For this, the user is presented with real-time feedback related to brain activity and must develop and optimize self-regulation strategies to improve the control performance (Strehl, 2014; Sitaram et al., 2017). Applications of neurofeedback range from performance optimization in sports (Mirifar et al., 2017) to clinical applications, where the main objective is to target abnormal functional structures to reduce or even eliminate symptoms (Thibault et al., 2018).

One field that can benefit from neurofeedback protocols is psychiatry (Kim and Birbaumer, 2014; Arns et al., 2017; Thibault et al., 2018). A common approach in these cases is affective neurofeedback (Linhartová et al., 2019), where the protocols target putative mechanisms of emotion regulation (Lindquist et al., 2012, 2016). Recent studies described promising results from neurofeedback training in patients with affective disorders, such as major depressive disorder (MDD) (Trambaiolli et al., 2021a). Moreover, recent studies show that neurofeedback training can lead to symptom improvements that last for weeks after intervention (Rance et al., 2018). Other observed benefits include the normalization of dysfunctional structures and plastic reorganization of intrinsic functional connectivity (Hampson et al., 2011; Scheinost et al., 2013) and directed effective connectivity (Zotev et al., 2011, 2013).

One crucial step for neurofeedback-based therapies is transferring the learned self-regulation strategies to real-life situations, outside of the overly controlled experimental setups (Brühl, 2015; Thibault et al., 2018). This approach is of particular importance to affective neurofeedback experiments since real-life scenarios most likely will include distractors, stressors, or other confounds that could affect the learned self-regulation strategies (Kadosh and Staunton, 2019). In this context, naturalistic setups are of extreme relevance to evaluate affective neurofeedback feasibility as a potential therapeutic approach. Although rare, neurofeedback and BCI experiments have been studied in the real-world (also referred to as “in-the-wild”) (Kosmyna, 2019). Moreover, virtual reality (VR) environments provide immersive, naturalistic experiences that can be used as an intermediate step toward applications in-the-wild. This approach is supported by computational (Renard et al., 2010) and instrumental (Cassani et al., 2020a) advances in the BCI field, providing the necessary tools to create neurofeedback protocols using VR.

This mini-review discusses the state-of-the-art of affective neurofeedback protocols under naturalistic conditions and open challenges in the field. For this, we first provide a brief overview of affective neurofeedback protocols using different neuroimaging modalities. We discuss the possibility of using VR as a transitional step toward experiments in-the-wild and evaluate current protocols using this feedback modality. Then, we assess the status of neurofeedback experiments outside the laboratory and discuss open challenges for developing affective neurofeedback protocols in such conditions.

## 2. Current Status of Affective Neurofeedback

Electroencephalography (EEG) was the first neuroimaging method used in developing affective neurofeedback protocols, with the first case reports dated from the 1990s (Rosenfeld et al., 1996; Baehr et al., 1997; Earnest, 1999). Usually, these protocols target the activity in prefrontal portions of the scalp. For example, the most common approach relies on possible asymmetries in channels over the prefrontal regions of the scalp (Choi et al., 2011; Peeters et al., 2014; Quaedflieg et al., 2016). This methodology assumes that the hyper- and hypo-activation of opposite hemispheres indicate the valence experienced during emotion regulation (Harmon-Jones et al., 2010) and may reflect symptoms in psychiatric patients (Thibodeau et al., 2006). Controlled experiments show that alpha asymmetry neurofeedback training may reduce negative mood and anxiety in healthy subjects (Quaedflieg et al., 2016; Mennella et al., 2017), as well as relieve depressive symptoms in patients with MDD (Choi et al., 2011; Wang et al., 2019). More examples of EEG-based neurofeedback include the control of alpha or beta bands over the parietal cortex (Escolano et al., 2014; Wang et al., 2019), sensorimotor rhythms (Lee et al., 2019), frequency ratios (Lee et al., 2019), among others.

With the advent of functional magnetic resonance imaging (fMRI), neurofeedback protocols were able to target more precisely the putative mechanisms of emotion regulation (Lindquist et al., 2012, 2016). In this context, different approaches were developed over the years, including protocols targeting the self-regulation of single regions of interest (ROIs) within these networks (Zotev et al., 2011; Young et al., 2014, 2017), multiple regions (Johnston et al., 2010, 2011; Linden et al., 2012; Mehler et al., 2018), or the functional connectivity between areas (Koush et al., 2017). In these cases, common targets include areas such as the lateral and medial prefrontal cortex (PFC), anterior cingulate cortex (ACC), insular cortex (IC), amygdala, among others (Linhartová et al., 2019). For instance, protocols targeting the amygdala self-regulation led to mood changes in healthy subjects (Zotev et al., 2011), and reduced symptoms in patients with MDD (Young et al., 2014, 2017). Moreover, functional connectivity reorganization was observed in these patients (Yuan et al., 2014; Young et al., 2018), as well as in subjects with post-traumatic stress disorder (PTSD) (Misaki et al., 2018). In approaches targeting multiple regions, although healthy subjects did not report changes in mood after training (Johnston et al., 2011), patients with MDD showed symptom improvement after multiple training sessions (Linden et al., 2012; Mehler et al., 2018), with benefits persisting at follow-up (Mehler et al., 2018).

The neurofeedback approach used by EEG and fMRI, although based on different methodologies, presents correlated neural mechanisms. For instance, the self-regulation of the amygdala during neurofeedback also engages brain structures from the frontal cortex (Zotev et al., 2013). Furthermore, studies with simultaneous EEG and fMRI recordings demonstrate an existing association between the amygdala self-regulation and the frontal EEG asymmetry (Zotev et al., 2016, 2018). In this context, Zotev and colleagues recently proposed a hybrid neurofeedback protocol, combining fMRI and EEG data during emotion regulation and demonstrating its feasibility with healthy subjects and MDD patients (Zotev et al., 2014, 2020; Zotev and Bodurka, 2020).

More recently, the use of functional near-infrared spectroscopy (fNIRS) has been explored (Ehlis et al., 2018; Kohl et al., 2020). Protocols targeting areas such as the dorsolateral PFC (dlPFC) (Marx et al., 2015; Hudak et al., 2017; Kimmig et al., 2019) and the orbitofrontal cortex (OFC) (Li et al., 2019) have been studied in healthy subjects and psychiatric populations, although not necessarily targeting emotion regulation. However, several studies using multivariate pattern recognition approaches demonstrated that fNIRS signals from the PFC are sufficient to decode affective processing during the visualization of pictures (Hosseini et al., 2011; Trambaiolli et al., 2018b, 2021b) and videos (Bandara et al., 2018; Hu et al., 2019), as well as during autobiographical affective imagery (Tai and Chau, 2009; Trambaiolli et al., 2018b, 2021b). In this context, Trambaiolli et al. (2018a) proposed a decoding-based affective neurofeedback protocol recalling positive autobiographical memories. Healthy participants were able to achieve satisfactory control of their prefrontal and occipital neural activity. However, further testing of this approach in clinical samples is still needed.

## 3. Virtual Reality as a Transitional Step

Virtual reality (VR) is an immersive three-dimensional graphical system that provides the sense of presence in the virtual world (Burdea and Coiffet, 2003). This sensation can be experienced by real-time interactions with the synthetic environment (Lotte et al., 2012). To improve the level of interaction, one may also use information from the participant, including body movements or physiological responses (Kritikos et al., 2021). This technology has been investigated as a potential therapeutic approach for neuromotor rehabilitation (Massetti et al., 2016; Ravi et al., 2017), or as an exposure therapy in psychiatry (Maples-Keller et al., 2017; Deng et al., 2019). Moreover, it also allows for investigations combining VR and neuromodulatory technologies (Cassani et al., 2020b). Even though the VR scenario emulates a real-world situation, the experimenter still has control over the environment, and the session can be stopped at any time the patient is uncomfortable (Maples-Keller et al., 2017). Thus, it provides advantages in experimental manipulation and control not previously available.

When combined with neurofeedback, VR can be used to evaluate how a less controlled environment influences the neurofeedback protocol, as well as the learned strategies. For instance, different neurofeedback protocols for attention training included a virtual classroom environment (Rohani and Puthusserypady, 2015; Hudak et al., 2017). In BCI protocols using EEG-based P300 signals, participants were able to control the system even in the presence of distracting elements (Rohani and Puthusserypady, 2015). In an fNIRS-based protocol, participants that learned how to self-regulate their hemodynamic signal in the dlPFC showed improved performance in inhibitory control tasks after training (Hudak et al., 2017). Moreover, a study comparing two-dimensional (2D) and three-dimensional (3D) VR feedback showed higher learning rates in the 3D-VR group (Berger and Davelaar, 2018).

Specific to affective neurofeedback, few protocols can be found in the literature using VR as a feedback tool. For instance, Lorenzetti et al. (2018) proposed a proof-of-concept fMRI-based experiment, with feedback provided by changing the color of the VR scenario. Participants were trained to self-regulate their neural activity in an ROI-based (amygdala) or decoding-based neurofeedback while inducing and sustaining complex emotions, such as tenderness and anguish. Although showing the technical feasibility of incorporating VR to affective neurofeedback, the color-changing scenario did not properly emulate a real-world situation.

On the other hand, the study from Aranyi et al. (2016) trained healthy participants to cheer up a virtual agent using the self-regulation of the asymmetry of fNIRS oxyhemoglobin concentrations in the dlPFC (this dataset is currently publicly available for future investigations; Charles et al., 2020). In a different study, Yamin et al. (2017) trained participants with depth electrodes in the amygdala to down-regulate this region while receiving feedback from virtual agents in a waiting room environment. In both experiments, participants were able to self-regulate the desired ROIs while receiving feedback in the VR setup. Moreover, the feedback provided is more consistent with possible situations faced by patients in real life.

## 4. Toward Affective Neurofeedback In-the-Wild

Neurofeedback protocols in-the-wild will allow for the continuity of the training outside of the research or clinical environment. This will ensure the reinforcement or adaptation of the developed strategies for the maintenance of long-term benefits.

MEG and fMRI have the highest spatial resolution among the most popular non-invasive neurofeedback protocols. Recent advances in the development of portable scanners (Boto et al., 2018; Wald et al., 2020; Zhang et al., 2020) bring hope to applications in the mid- and long-term future. However, the physical restraints associated with current equipment make in-the-wild experiments impractical (Sulzer et al., 2013). In this context, to increase the portability of a neurofeedback system, the use of mobile or wireless EEG and fNIRS devices emerge as a possible solution (Hondrou and Caridakis, 2012; Pinti et al., 2018; Quaresima and Ferrari, 2019). Comparative studies show that mobile EEG devices have similar accuracy to over the bench equipment (De Vos et al., 2014; Ries et al., 2014), and its applications include studying neural correlates of motor behavior (Packheiser et al., 2020), attention monitoring (Ladouce et al., 2019), and mental state monitoring in ambulatory conditions (Albuquerque et al., 2020; Parent et al., 2020). However, these devices require more pre-processing and filtering steps to compensate for biological artifacts (for instance, ocular and muscular activity) or instrumental and environmental noises (electrode misplacement, electrical, and radio-frequency interference, among others) (Fairclough and Lotte, 2020). Mobile fNIRS devices have also been tested in-the-wild, for instance, while walking (Doi et al., 2013; Mirelman et al., 2014; Maidan et al., 2016), playing table tennis (Balardin et al., 2017b), or playing violin (Vanzella et al., 2019). Similar to EEG, mobile fNIRS have to account for muscular artifacts (Balardin et al., 2017a), as well as instrumental and environmental noises such as optical decoupling and local luminance (Pinti et al., 2018). These approaches can also be combined into hybrid protocols, which have the advantage of combining multiple aspects of neural activity (for example, electric and hemodynamic changes) (Pfurtscheller et al., 2010; Müller-Putz et al., 2011, 2015). In this context, researchers have been developing mobile, and modular multimodal sensors combining EEG and fNIRS channels (von Lühmann et al., 2016).

Studies investigating the feasibility of affective neurofeedback protocols in-the-wild are still rare. However, studies using similar approaches in-the-wild, such as brain-machine interfaces (BMI), can be found in the literature. For instance, healthy participants were able to control the directions of a wheelchair based on P300 (Iturrate et al., 2009), steady-state visual evoked potentials (SSVEP) (Müller et al., 2013), or P300+SSVEP signals combined (Li et al., 2013). Moreover, using a stimulus-independent design, paraplegic patients learned to control a lower limb exoskeleton during gait using a motor imagery protocol (Donati et al., 2016). These examples provide a rationale for the possibility of experimenting with affective neurofeedback protocols in-the-wild.

## 5. Challenges for Affective Neurofeedback In-the-Wild

Although presenting promising results in controlled environments, current affective neurofeedback protocols face several methodological challenges for real-world applications (Kosmyna, 2019). If neglected, these aspects will become a barrier for in-the-wild setups, and cause frustration and lead to discontinued training. Due to these caveats, some of these challenges are listed, and potential solutions are discussed next.

### 5.1. Convenience

The physical limitations experienced during current protocols play a fundamental challenge for naturalistic experiences. For instance, fMRI protocols are physically restrictive, and the user may experience claustrophobia during the session (Sulzer et al., 2013). On the other hand, EEG- and fNIRS-based protocols may require a relatively long time for the cap preparation (positioning, conductive gel, calibration, among others). It also results in residual gel over the participant's head after the session. In this context, the use of dry and active electrodes is a possible alternative to reduce the inconvenience of the setup preparation (Lopez-Gordo et al., 2014). For instance, commercially available EEG headbands using dry electrodes were successfully employed in emotion classification in the lab (Arsalan et al., 2019), as well as during attention training neurofeedback (Kovacevic et al., 2015) and stress monitoring (Parent et al., 2020) experiments in-the-wild. Thus, these types of setups should be further explored for affective neurofeedback in-the-wild.

### 5.2. Feedback Modality

The feedback modality is important during reinforcement learning. For studies using VR, visual feedback seems to be an obvious choice. However, for in-the-wild applications, the best feedback approach is still an open question. One portable option would be using a laptop for real-time data processing and providing visual feedback, but other mobile devices, such as cellphones and tablets, should be explored. For instance, stimulus-dependent BCI protocols are already possible using these devices (Wang et al., 2013; Jijun et al., 2015). Although not typical for affective neurofeedback, other feedback modalities should be explored in-the-wild (Sitaram et al., 2017). For instance, haptic and auditory feedback could be integrated with current wearable technologies, such as smartwatches and headphones, respectively.

### 5.3. Attention and Workload Variations

Under naturalistic conditions, the neurofeedback protocol will need to consider different effects caused by the environment. For instance, environmental distractions may influence the attention, stress levels, and mental workload, leading to physiological noises such as abrupt changes in EOG and EMG signals caused by reflexive eye movements or muscular responses (Theeuwes et al., 1998; de Wied et al., 2006). Moreover, our brain is continuously processing environmental information, so involuntary neural patterns will also be caused by the environment (Engelien et al., 2000; Boly et al., 2004). As previously mentioned, multimodal approaches could be a solution combining biosignals from different sources to separate the desired neural signal from physiological noises (Pfurtscheller et al., 2010; Müller-Putz et al., 2011, 2015). Additionally, hybrid systems may be used to monitor levels of stress and mental workload to adapt the neurofeedback algorithm (Albuquerque et al., 2020; Parent et al., 2020). For instance, the study from Falk et al. (2010) evaluated the effects of physiological and auditory noises emulating real-world situations during tasks commonly used in BCI experiments. After using compensatory algorithms, participants were able to control the BCI system with performances similar to the silent conditions. In a different scenario, the incorporation of error-related potentials in the BCI algorithm also led to optimized training results (Chavarriaga et al., 2014; Spüler and Niethammer, 2015).

### 5.4. Algorithm Robustness

As previously described, EEG and fNIRS are the most commonly used neuroimaging modalities in-the-wild. However, these technologies are prone to non-neural physiological noises, such as electromagnetic fields for EEG and environmental light sources for fNIRS (Fatourechi et al., 2007; Strait and Scheutz, 2014; Minguillon et al., 2017). Such noise sources require robust artifact removal algorithms (Fairclough and Lotte, 2020), for example using adaptative filters (Rosanne et al., 2021), or real-time independent component analysis (ICA) (Mayeli et al., 2016; Val-Calvo et al., 2019). Additionally, these algorithms should be simple and computational cost-effective, once they will ultimately be implemented and run in portable devices.

### 5.5. Decoding Performance

Some neurofeedback protocols use decoding-based approaches (Taschereau-Dumouchel et al., 2020), which allow the bi-directional (up and down) self-regulation of multiple regions or channels, leading to differential plasticity changes in specific subgroups of neurons (Shibata et al., 2019). This approach may require multiple training/calibration blocks before the algorithm starts to identify the user's neural patterns. Although a recent survey shows that while the participants are comprehensive about the need for training blocks during the first session (Kosmyna, 2019), it may become a problem for long-term usage. Also, under naturalistic setups, the decoder is likely to present higher intra-session variability given the shifts in attention and stress levels, mental workload, or environmental noises, as mentioned above. In this context, alternatives such as the use of subject-independent protocols (Ray et al., 2015; Trambaiolli et al., 2021b), artificially generated training samples (Lotte, 2015), or self-recalibrating classifiers (Bishop et al., 2014) have been explored. Moreover, these approaches will be facilitated given recent efforts to create open-access big-databases from decoding-based neurofeedback (Cortese et al., 2021), or affective decoding experiments (Abadi et al., 2015; Lan et al., 2020).

### 5.6. Non-specific Effects

Although showing potential benefits in specific clinical populations, the results from neurofeedback training can be driven by non-specific effects (Ros et al., 2020). For instance, similar to other clinical interventions, patients under neurofeedback training may present clinical improvement due to placebo effects (Thibault and Raz, 2017; Thibault et al., 2017). Also, other areas composing large-scale networks may present variations in addition to the targeted region of interest (Mayeli et al., 2020). In the context of protocols under naturalistic conditions, the participant may show improvement in mood and anxiety symptoms based on the enriched environment or interaction with the high-tech setup. In this context, studies investigating affective neurofeedback in naturalistic setups must follow proper designs for control groups and measures (Sorger et al., 2019), and adequate reporting practices (Ros et al., 2020).

### 5.7. Replicability and Reproducibility

Current neurofeedback studies still lack a detailed description of online signal processing (Heunis et al., 2020), while recent checklists do not address the detailed description of offline analysis (Tursic et al., 2020). The accurate and detailed description of experimental protocols and analytical methods is crucial to ensure proper replication studies in the neurofeedback field (Melnikov, 2021). This is particularly important for experiments in-the-wild that will require extensive pipelines to deal with new types of movement and environmental noises (Fairclough and Lotte, 2020).

### 5.8. Neurofeedback Illiteracy

BCI illiteracy is a phenomenon in which 10–50% of BCI users will not gain voluntary control of their neural activity (Allison and Neuper, 2010; Edlinger et al., 2015). This concept can also be expanded to neurofeedback protocols (Alkoby et al., 2018). In this context, there is an increasing interest for physiological or psychological predictors describing who would benefit from neurofeedback training (Alkoby et al., 2018). For example, several studies report functional neural networks (Weber et al., 2011; Scheinost et al., 2014; Wan et al., 2014; Trambaiolli et al., 2018a) and neuroanatomical differences (Halder et al., 2013) are related to the neurofeedback literacy in both healthy and clinical populations. Also, psychological aspects as control belief, frustration, concentration, among others, play an essential role during the training performance (Alkoby et al., 2018; Kadosh and Staunton, 2019). However, studies of illiteracy predictors are mainly based on experiments under very controlled environments. How these predictors will be affected during neurofeedback training in naturalist setups remains unknown. Moreover, future investigations should identify possible differences between responders in controlled environments and responders under naturalistic setups. For instance, those undergoing VR-based training might experience cybersickness (Weech et al., 2019), which may lead to a new illiteracy category. This way, proper training, and transfer strategies will be developed for each group of users.

## 6. Conclusion

Affective neurofeedback has been investigated as a potential therapeutic tool in psychiatry, showing promising results in many clinical samples. To advance this technology, the study of neurofeedback protocols under naturalistic conditions is necessary. It will optimize the transference of the learned self-regulation strategies to real-world scenarios or extend the neurofeedback training outside the lab. This mini-review showed that VR setups are already being explored for affective neurofeedback protocols and can be used as a transitional step before investigations in-the-wild. Moreover, the rise of portable EEG and fNIRS devices and the successful application of these instruments for BCI protocols in-the-wild endorse the technical feasibility of affective neurofeedback in such conditions. Besides improving the strategy transference and continuous training, designs that work in-the-wild could be a solution to bring neurofeedback to patients who can not visit large imaging centers for different reasons (location, mobility limitations, among others). Moreover, these protocols and related training can be expanded to non-clinical environments, including applications in gaming, affective computing, sports performance, among others. We close with a discussion on open challenges for neurofeedback training in-the-wild, including: convenience and reliability of the neurofeedback setup, environmental effects in attention and workload, non-specific effects, and possible new neurofeedback illiteracy categories.

## Author Contributions

All authors contributed equally to the intellectual efforts and writing of the paper.

## Conflict of Interest

The authors declare that the research was conducted in the absence of any commercial or financial relationships that could be construed as a potential conflict of interest.
